# Galveston County Medical Society, Minutes

**Published:** 1894-01

**Authors:** David Cerna

**Affiliations:** Secretary


### Society Notes.

### GALVESTON COUNTY MEDICAL SOCIETY.

A regular meeting of the Galveston County Medical Society was held at the Medical College building, on the 4th of December, 1893, at 8 o'clock p. m.

The President, Dr. William Keiller, in the chair.

Present: Members—Keiller, Sampson, Thompson, Haden, Gwyn, Flavin, Hendricks, Barrell, Hulen, Blue, Lee and Cearna, Guests—Drs. B. E. Hadra, J. W. Nixon and C. F. McClendon. Medical students—Magnenar, Allen, Thompson, Sampson and Davidson.

The minutes of the last meeting were read and approved. Dr. William Keiller then read a paper entitled "Uterine Hemorrhages," which elicited a considerable discussion, in which Drs West, Sampson and Thompson took part.

The paper was referred to the Publishing Committee.

On motion, the reading of Dr. Lee's paper, "Vomiting in Pregnancy," was, owing to the lateness of the hour, postponed till the next meeting.

Dr. J. M. Gray was unanimously elected a member of the Society.

The election of a new treasurer being in order, balloting was carried out, Drs. Gray and Cerna being the only two candidates by nomination of the members present. Ten votes were cast, 7 to 3 in favor of Dr. Gray, this gentleman being, therefore, declared treasurer of the Society, to fill the vacancy caused by the resignation of Dr. W. F. Baldinger.

Dr. West nominated Dr. B. E. Hadra as member of the Society. The nomination was seconded by Dr. Cerna.

The President, then appointed Dr. James Kennedy as one of the essayists for the next meeting.

On motion, the Society adjourned.

DAVID CERNA, M. D., Secretary.

